# Effect of muscle strengthening exercise and time since onset in patients with amyotrophic lateral sclerosis: A 2-patient case series study

**DOI:** 10.1097/MD.0000000000011145

**Published:** 2018-06-22

**Authors:** Naoki Kato, Goichi Hashida, Kuni Konaka

**Affiliations:** a Department of Rehabilitation, Osaka University Medical Hospital; b Department of Neurology, Osaka University Graduate School of Medicine, Suita, Osaka; c Department of Physical Therapy, Faculty of Health Science, Osaka Yukioka College of Health Science, Ibaraki, Osaka, Japan.

**Keywords:** amyotrophic lateral sclerosis, physical therapy, resistance training, time since onset

## Abstract

Exercise for patients with amyotrophic lateral sclerosis (ALS) can be expected to improve function at the early stage of disease, but improvement cannot be expected at the late stage. However, no report has investigated the correlation between the effect of exercise and time since onset. This study examined the relevance between the effect of muscle strengthening exercise and time since onset in patients with ALS.

This study is a retrospective nonconsecutive case series study at a single university hospital. We included 2 patients with ALS who were admitted twice. Case 1 was a 60-year-old man with the bulbar type. He was hospitalized 10 months (ALS functional rating scale-revised, 42 points) and 1 year and 8 months (33 points) after onset. Case 2 was a 52-year-old man with the lower limb type. He was hospitalized 1 year and 3 months (44 points) and 1 year and 8 months (33 points) after onset. Physical therapy was implemented daily on weekdays for approximately 30 minutes. The intervention period was 2 weeks. Lower limb muscle strengthening exercises were mainly performed and exercise intensity was adjusted to a modified Borg Scale score of 5 (lower limbs). The study investigated knee extension muscle strength (KEMS) by using a hand-held dynamometer and Functional Ambulation Categories (FAC) at the start and end of physical therapy during each hospitalization.

KEMS improved during the initial hospitalization in both patients, and FAC improved in Case 2; neither KEMS nor FAC improved during rehospitalization in either patient. In Case 1, KEMS was maintained for 10 months.

The current results suggest that the positive effect of muscle strengthening exercise is greater at the early stage and may be maintained in patients with bulbar type ALS. In addition, improvement can be achieved approximately 1 year after onset and in patients with an ALSFRS-R score of 40 points or more. Therefore, it is necessary to initiate and continue exercise earlier after onset.

## 1 Introduction

Amyotrophic lateral sclerosis (ALS) is a neurodegenerative disease in which the cell bodies of both upper and lower motor neurons degenerate rapidly. Muscle weakness, spasticity, respiratory failure, and communication disorders are the primary clinical symptoms, and activity and social participation are restricted. Expected survival is 2 to 4 years on average, and most patients die due to respiratory failure. Riluzole (Sanofi-Aventis) and edaravone (Mitsubishi Tanabe Pharma) are approved in Japan as drug therapy for ALS. However, symptomatic treatment and rehabilitation are important because there is no curative treatment available.^[1,2]^

Generally, exercise for patients with ALS can be expected to improve function at the early stage of disease, but improvement cannot be expected at the late stage.^[1,3]^ However, no report has investigated the correlation between the effect of exercise and time since onset, and the period or severity at which the clinical effect can be expected remains unclear.

It is difficult to compare patients with ALS due to multiple disease types and the variable rate of progression for each patient.^[2]^ Therefore, this report aimed to demonstrate the effect of muscle strengthening exercise and time since onset in patients with ALS who were hospitalized twice.

## 2 Methods

### 2.1 Ethical consideration

This study was approved by the Osaka University Medicine Hospital Ethics Committee. This research plan was published on the web (www.hosp.med.osaka-u.ac.jp/research/data/rehabilitation1.pdf), and informed consent was obtained orally and opted out based on the provisions of the Ethics Committee. Personal information was made anonymous.

### 2.2 Patients

This study is a retrospective nonconsecutive case series study at a single university hospital. We included 2 patients with ALS who were admitted to the Osaka University Medical Hospital twice and underwent physical therapy from April 2015 to December 2016. Patients who were not ambulatory and admitted for the treatment of other complications (such as infection) were excluded.

*Case 1*. The patient was a 60-year-old man, with a height of 161 cm, weight of 61 kg, and bulbar type ALS. He developed hoarseness and dysarthria. He was hospitalized 10 months after onset. He was diagnosed as having ALS, and began treatment with riluzole and biphasic positive airway pressure at night. He was rehospitalized to receive edaravone intravenous drip at 1 year and 8 months after onset. At rehospitalization, his weight was decreased to 50 kg. Regarding complications and medical history, he had a history of left knee joint surgery due to a motor vehicle collision and was diagnosed as having lumbar spondylosis 4 months after onset of ALS. Neurological symptoms and disorders of activities of daily living due to these diseases were not observed.

*Case 2*. The patient was a 52-year-old man, with a height of 171 cm, weight of 61 kg, and lower limb type ALS. He developed gait disturbance, and was hospitalized 1 year and 3 months after onset. He was diagnosed as having ALS and began taking riluzole. He was rehospitalized to receive edaravone intravenous drip 1 year and 8 months after onset. No notable complications or medical history was observed.

Both patients were diagnosed based on the Awaji criteria.^[4]^ No rehabilitative intervention was provided between the initial hospitalization and rehospitalization.

### 2.3 Interventions

Physical therapy was generally implemented daily on weekdays for approximately 30 minutes by a skilled physical therapist who was specialized in neuromuscular disorders. The intervention period was 2 weeks. Lower limb muscle strengthening exercises were performed for approximately half of the session, and bicycle ergometer, respiratory exercises, and gait exercises were also performed. Muscle strengthening exercises were resistance training using weights and machines. Exercise intensity was adjusted to a modified Borg Scale^[5]^ score of 5 (lower limbs).^[1,3]^ Muscle strengthening exercises, respiratory exercises, and gait exercises were instructed to be performed as self exercises after discharge.

### 2.4 Methods

The study investigated knee extension muscle strength (KEMS) and Functional Ambulation Categories (FAC)^[6]^ at the start and end of physical therapy during each hospitalization. The data were collected based on medical records. KEMS was measured 3 times by using a hand-held dynamometer (μ-Tas F-1, ANIMA) as described^[7]^ and was standardized. The rate of improvement was also calculated based on the following formulae:

KEMS (Nm/kg) = (average of 3 attempts) (N) × lower leg length (m)/body weight (kg)

Improvement rate (%) = ((KEMS at end) − (KEMS at start))/(KEMS at start) × 100

## 3 Results

**Figure 1:**
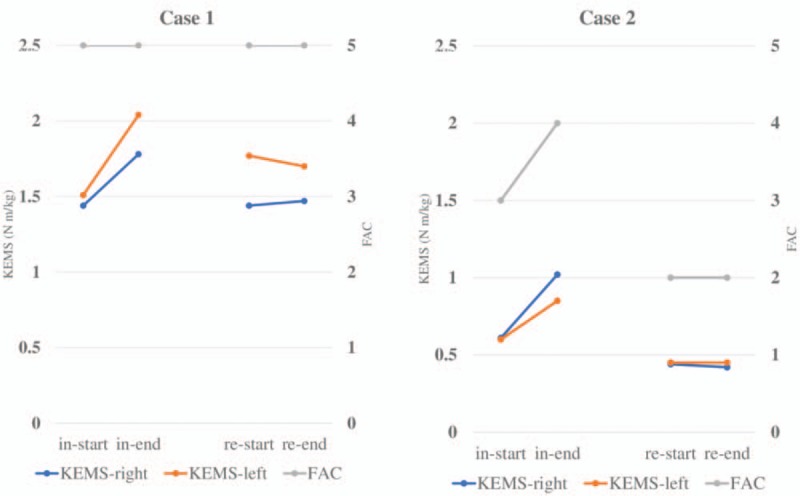
Knee extension muscle strength and Functional Ambulation Categories score over time. FAC = Functional Ambulation Categories; in-end, end of physical therapy during the initial hospitalization; in-start, start of physical therapy during the initial hospitalisation, KEMS = knee extension muscle strength; re-end, end of physical therapy during rehospitalization; re-start, start of physical therapy during rehospitalization.

Table 1 shows physical function, as evaluated using percent forced vital capacity, cough peak flow, grip strength, and ALS functional rating scale-revised (ALSFRS-R),^[8]^ at the start of physical therapy, and Figure 1 presents the KEMS and FAC scores over time. There were no missing data for any of the variables. No adverse events were observed during the hospitalization.

**Table 1:**
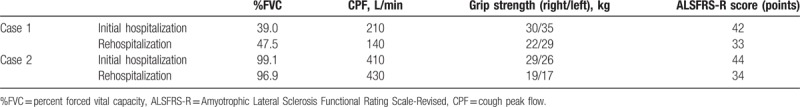
Data of physical function in both patients.

KEMS improved by 20% or more, which exceeded the minimal clinically important difference (MCID),^[9]^ during the initial hospitalization in both patients; in Case 2, the FAC score improved from 3 points to 4 points, and the patient developed independent gait. In contrast, neither KEMS nor the FAC score improved during rehospitalization in either patient.

In Case 1, KEMS at the start of the initial hospitalization was maintained until 10 months later. In Case 2, KEMS decreased by approximately 25% at rehospitalization compared with the start of the initial hospitalization.

## 4 Discussion

### 4.1 The effect of muscle strengthening exercise and time since onset

In both patients, KEMS improved during the initial hospitalization, but it did not improve during rehospitalization. However, in Case 1, KEMS exceeded the normal value (1.3–1.4 Nm/kg),^[10]^ but in Case 2, it was below the normal value. Thus, different physiological mechanisms are inferred.

In ALS, motor neurons degenerate rapidly. Residual motor neurons expand motor units by collateral sprouting; thus, the decrease of motor unit number is compensated for and muscle strength is maintained in patients with ALS at the early stage.^[11]^ In Case 1, KEMS did not change between the initial hospitalization and rehospitalization, but cough peak flow, grip strength, and ALSFRS-R score were decreased. Therefore, it is inferred that KEMS was maintained by collateral sprouting of residual motor neurons. As a physiological mechanism of muscle strengthening, the effect of neurological factors, such as recruitment of muscle fibers, is larger during the initial weeks after exercise.^[12]^ Thus, the positive effect of muscle strengthening exercise due to a neural factor can be expected during the initial hospitalization because more motor neurons would survive even with the same muscle strength. The authors consider that this mechanism improved KEMS during the initial hospitalization in Case 1.

Muscle weakness in patients with ALS is thought to be caused by disease progression and disuse muscle weakness.^[1]^ Exercise for ambulatory patients with motor neuron disease (ALS, and spinal and bulbar muscular atrophy) is more effective when muscle strength or function are lower; this suggests an improvement in disuse muscle weakness.^[13–15]^ However, as muscle weakness progresses, it also becomes an overload in activities of daily living, and improvement cannot be expected.^[1]^ Case 2 was ambulatory during the initial hospitalization. Therefore, the authors consider that disuse muscle weakness was improved, which led to improvement in the FAC score and independent gait.

These current results suggest that a positive effect of muscle strengthening exercise can be expected earlier after disease onset, and gait ability can improve. KEMS and gait ability improved during the initial hospitalization (approximately 1 year after onset, ALSFRS-R score of 40 points or more), but they did not improve during rehospitalization (1 year and 8 months after onset, ALSFRS-R score < 35 points) in both patients. It has been reported that most patients with ALS are diagnosed at 1 year and 2 months after onset and with an ALSFRS-R score of 39 points^[16]^; thus, the period during which a positive effect can be expected may be very short. Therefore, despite disease type, muscle weakness, and gait disturbance, it is important to initiate exercise for maintaining or improving KEMS and gait ability as soon as patients are diagnosed as having ALS.

### 4.2 The course of effect of muscle strengthening exercise

In Case 2, KEMS was decreased by approximately 25% at rehospitalization (5 months later) compared with the start of the initial hospitalization. The gain of muscle strengthening exercise during the initial hospitalization was thought to be lost at rehospitalization because the measured value (not standardized) of KEMS in patients with ALS is decreased by approximately 35% 1 year after diagnosis.^[17]^ However, in Case 1, it was maintained up to rehospitalization (10 months later), and the measured value of KEMS exceeded natural history.^[17]^ Although the period during which the gain of exercises for lower limb muscle strength is maintained remains unclear, one report stated that the gain had been lost 2 months after intervention.^[18]^ The current results demonstrate that patients with ALS can maintain the positive effect of muscle strengthening exercise for nearly 1 year.

The patient in Case 1 had bulbar type disease. The half-life of the motor unit number is approximately 1.7 times longer at the noninitial site of onset than at the initial site of onset in patients with ALS.^[19]^ In Case 1, knee extensor muscles were presumed to be innervated by remaining motor neurons because of moderate disease progression. Therefore, the authors consider that self-exercise could be continued because of normal KEMS and independent gait; therefore, the positive effect was maintained. In other words, exercise should be continuously performed to maintain the effect of muscle strengthening exercise in patients with bulbar type ALS.

### 4.3 Limitations

The sample size was very small and the study did not use a statistical method. It may not be possible to detect any improvements below the MCID because of low measurement accuracy of the hand-held dynamometer. It is hypothesized that the effect of muscle strengthening exercise is dependent on the degree of collateral sprouting by residual motor neurons and disuse muscle weakness in patients with ALS. Therefore, for example, the positive effect may be smaller during the early stage, when comparing the period in which muscle strength is maintained at a normal value by collateral sprouting and disuse muscle weakness is beginning to appear.

## 5 Conclusions

It is suggested that a positive effect of muscle strengthening exercise can be obtained during the early stage of ALS despite muscle weakness or gait disturbance. In addition, improvement can be achieved approximately 1 year after onset and in patients with an ALSFRS-R score of 40 points or more. The positive effect of muscle strengthening exercises for knee extensor muscles and gait ability can be maintained in patients with bulbar type ALS. Therefore, it is necessary to initiate and continue exercise for the knee extensor muscles as soon as patients are diagnosed, in order to maintain and improve gait ability. Further studies with a larger number of patients and statistical analyses are required to determine the duration of the disease and other factors that influence the effect of muscle strengthening exercise on ALS.

## Acknowledgment

We would like to thank Editage (www.editage.jp) for English language editing.

## Author contributions

**Conceptualization:** Naoki Kato.

**Data curation:** Naoki Kato.

**Formal analysis:** Naoki Kato.

**Investigation:** Naoki Kato.

**Methodology:** Naoki Kato.

**Supervision:** Goichi Hashida, Kuni Konaka.

**Writing – original draft:** Naoki Kato.
